# Transforming growth factor beta 1 is associated with subclinical carotid atherosclerosis in patients with systemic lupus erythematosus

**DOI:** 10.1186/s13075-023-03046-2

**Published:** 2023-04-17

**Authors:** Fuensanta Gómez-Bernal, Juan Carlos Quevedo-Abeledo, María García-González, Yolanda Fernández-Cladera, Agustín F. González-Rivero, Candelaria Martín-González, Miguel Á. González-Gay, Iván Ferraz-Amaro

**Affiliations:** 1 Division of Central Laboratory, Hospital Universitario de Canarias, Tenerife, Spain; 2 Division of Rheumatology, Hospital Doctor Negrín, Las Palmas de Gran Canaria, Spain; 3 Division of Rheumatology, Hospital Universitario de Canarias, Tenerife, Spain; 4 Division of Internal Medicine, Hospital Universitario de Canarias, Tenerife, Spain; 5 Department of Internal Medicine, University of La Laguna (ULL), Tenerife, Spain; 6 Epidemiology, Genetics and Atherosclerosis Research Group On Systemic Inflammatory Diseases, Hospital Universitario Marqués de Valdecilla, IDIVAL, Santander, Spain; 7 Division of Rheumatology, Hospital Universitario Marqués de Valdecilla, Universidad de Cantabria, Santander, Spain; 8 Cardiovascular Pathophysiology and Genomics Research Unit, School of Physiology, Faculty of Health Sciences, University of the Witwatersrand, Johannesburg, South Africa; 9 Division of Rheumatology, IIS-Fundación Jiménez Díaz, Madrid, Spain

**Keywords:** Transforming growth factor beta, Systemic lupus erythematosus

## Abstract

**Background:**

Transforming growth factor beta (TGF-β1) is a multifunctional cytokine that has anti-inflammatory and immunosuppressive effects. TGF-β1 has been linked to cardiovascular disease in the general population. The immunosuppressive effect of TGF-β1 is believed to be dysregulated in patients with systemic lupus erythematosus (SLE). In the present work, we aimed to study the relationship of serum levels of TGF-β1 with subclinical carotid atherosclerosis in patients with SLE.

**Methods:**

The study included 284 patients with SLE. Serum levels of TGF-β1 and subclinical carotid atherosclerosis (by carotid ultrasonography) were evaluated. In addition, the complete lipid profile and insulin resistance were analyzed. Multivariable linear and logistic regression analysis was performed to establish the relationship of TGF-β1 with carotid subclinical atherosclerosis adjusting for traditional cardiovascular risk factors that included lipid profile and insulin resistance.

**Results:**

Circulating TGF-β1 was positively and significantly associated with higher levels of LDL:HDL cholesterol ratio and atherogenic index. TGF-β1 was also associated with significantly lower levels of HDL cholesterol and apolipoprotein A1. Remarkably, TGF-β1 was associated with the presence of carotid plaque not only after adjustment for demographics (age, sex, body mass index, diabetes, hypertension, and aspirin use) but also after adjustment for relationships of TGF-β1 with lipid profile molecules, insulin resistance, and SLEDAI disease score (odds ratio 1.14 [95% confidence interval 1.003–1.30], *p* = 0.045).

**Conclusion:**

TGF-β1 serum levels are positively and independently associated with the presence of subclinical atherosclerosis disease in patients with SLE.

**Supplementary Information:**

The online version contains supplementary material available at 10.1186/s13075-023-03046-2.

## Background

Transforming growth factor beta 1 (TGF-β1) is a polypeptide member of the TGF-β superfamily of cytokines. It is a secreted protein that performs many cellular functions, including the control of cell growth, cell proliferation, cell differentiation, and apoptosis. The immunological function of TGFβ1 includes multifunctional anti-inflammatory and immunosuppressive effects [1]. However, TGF-β1 has also been linked to inflammation and autoimmunity in combination with certain cytokines and T cells [2]. In addition to TGFβ1, TGF-β has two other isoforms, TGF-β2 and TGF-β3. However, cells of the immune system mainly produce TGF-β1, which is the isoform usually found in the plasma [3].

Systemic lupus erythematosus (SLE) is a chronic autoimmune disease of unknown cause that can affect virtually any organ of the body. Immunologic abnormalities, especially the production of various antinuclear antibodies, are typical of this condition. Patients with SLE exhibit a variable spectrum of clinical features ranging from mild skin and joint involvement to life-threatening renal, hematologic, or central nervous system manifestations. A higher prevalence of atherosclerosis is present in patients with SLE. A systematic review found that the risk of cardiovascular disease (CVD) (including myocardial infarction, cerebrovascular disease, and peripheral vascular disease) among patients with SLE is at least double compared to the general population [4]. Accelerated atherosclerosis in SLE has been attributed to the damage caused by the disease [5], which will eventually lead to the presence of an altered lipid profile [6–10] and the appearance of insulin resistance and beta cell dysfunction [11–13].

CVD in the general population has been associated with TGF-β1 signaling pathways. Plasma levels of TGF-β1 were found to be reduced in patients with advanced atherosclerosis and angiographically proven coronary artery disease [14–16]. In addition, genetic mutations in TGF-β pathway genes and dysregulation of signaling have emerged as an important molecular pathway involved in CVD in adults [17]. TGF-β1 expression in patients with SLE was found to be dysfunctional [18]. In this sense, the production of TGF-β1 by the lymphocytes of patients with SLE is reduced compared to controls [19], while the production of autoantibodies in SLE can be abolished by treatment with TGF-β [20].

In the present study, we have evaluated serum TGF-β1 levels in a large series of SLE patients in whom traditional CVD risk factors, including lipid profiles and insulin resistance, have been fully characterized. Our objective was to analyze the relationship of TGF-β1 with the presence of subclinical carotid atherosclerosis disease adjusting for these classic CVD risk factors.

## Material and methods

### Study participants

This was a cross-sectional study that included 284 patients with SLE. All SLE patients were 18 years old or older, had a clinical diagnosis of SLE, and fulfilled ≥ 4 American College of Rheumatology (ACR) classification criteria for SLE [21]. They had been diagnosed by rheumatologists and were periodically followed up at rheumatology outpatient clinics. Patients taking prednisone, at an equivalent dose of ≤ 10 mg/day, were allowed to participate, as glucocorticoids are often used in the treatment of SLE. Patients were excluded if they had a history of myocardial infarction, angina, stroke, cancer, and/or any other chronic diseases or evidence of active infection. The research was carried out in accordance with the Declaration of Helsinki. The study protocol was approved by the Institutional Review Committee at Hospital Universitario de Canarias and at Hospital Universitario Doctor Negrín (both in Spain), and all patients provided informed written consent (Approval Number 2015_84).

### Data collection and laboratory assessments

Individuals included in the study completed a cardiovascular risk factor and medication use questionnaire and underwent a physical examination. Weight, height, body mass index (BMI), abdominal circumference, and systolic and diastolic blood pressure (measured with the participant in a supine position) were assessed under standardized conditions. Information regarding smoking status (current smokers) and hypertension treatment was obtained from the questionnaire. Medical records were reviewed to ascertain specific diagnoses and medications. Fasting serum samples were collected and frozen at − 80 °C until analysis of circulating lipids. Cholesterol, triglycerides, and HDL cholesterol were measured using the enzymatic colorimetric assay (Roche). LDL cholesterol was calculated using the Friedewald formula. The atherogenic index was calculated using Castelli’s formula (total cholesterol/HDL cholesterol). A standard technique was used to measure high-sensitivity C-reactive protein (CRP). The homeostatic model assessment (HOMA) method was performed to determine IR. Briefly, the HOMA model enabled an estimate of insulin sensitivity (%S) and β cell function (%B) from fasting plasma insulin, C-peptide, and glucose concentrations. In this study, we used HOMA2, the updated computer HOMA model [22]. This model can be used to assess insulin sensitivity and beta cell function from paired fasting plasma glucose and specific insulin, or C-peptide, concentrations across a range of 1–2200 pmol/l for insulin and 1–25 mmol/l for glucose. C-peptide better estimates β cell function since it is a marker of secretion, and insulin data is preferable when calculating %S since HOMA-%S is derived from glucose disposal as a function of insulin concentration. In our study, IR and %S were calculated using insulin serum levels. Otherwise, %B was calculated using C-peptide serum levels. The computer model provided a value for insulin sensitivity expressed as HOMA2-%S (in which 100% is normal). HOMA2-IR (insulin resistance index) is simply the reciprocal of %S. SLE disease activity and damage were assessed using the Systemic Lupus Erythematosus Disease Activity Index-2000 (SLEDAI-2 K) [23] and the Systemic Lupus International Collaborating Clinics/American College of Rheumatology (SLICC/ACR Damage Index-SDI) [24], respectively. For the purpose of the present study, the SLEDAI-2 k index was broken down into none (0 points), mild (1–5 points), moderate (6–10 points), high (11–19), and very high activity (> 20) as previously described [25]. Disease severity was measured as well, using the Katz Index [26]. An ELISA kit was used for the detection of TGF-β1 (Elabscience, USA). A standard calibration curve was made on each ELISA plate using duplicate controls and blanks. Both intra- and inter-coefficients of variability are < 10% for this assay.

### Carotid ultrasound assessment

A carotid ultrasound examination was performed to assess cIMT in the common carotid artery and to identify focal plaques in the extracranial carotid tree in patients with SLE [27]. A commercially available scanner, the Esaote Mylab 70 (Genoa, Italy), equipped with a 7–12-MHz linear transducer and an automated software-guided radiofrequency technique, quality intima media thickness in real-time (QIMT, Esaote, Maastricht, Holland), was used for this purpose. As previously reported [28], based on the Mannheim consensus, the plaque criteria in the accessible extracranial carotid tree (common carotid artery, bulb, and internal carotid artery) were defined as follows: a focal protrusion in the lumen measuring at least cIMT > 1.5 mm, a protrusion at least 50% greater than the surrounding cIMT, or arterial lumen encroaching > 0.5 mm [29].

### Statistical analysis

Demographic and clinical characteristics in patients with SLE and controls were described as mean ± standard deviation (SD) or percentages for categorical variables. For non-normally distributed continuous variables, data were expressed as median and interquartile range (IQR). The relation of CV features of the disease with circulating TGF-β1 was assessed through multivariable linear and logistic regression analysis. The confounders were selected from the demographics-related data that had a univariable relationship with TGF-β1 with a *p* value less than 0.20. Additionally, to neutralize the effect of other modifications on the lipid pattern, an additional multivariable analysis was constructed, adding to the model those differences in lipid-related molecules between patients and controls with a *p*-value < 0.20. All the analyses used a 5% two-sided significance level and were performed using the Stata software, version 17/SE (StataCorp, College Station, TX, USA). *p*-values < 0.05 were considered statistically significant.

## Results

### Demographics and disease-related data of systemic lupus erythematosus patients

Demographic and disease-related characteristics of patients with SLE included are shown in Table 1. Most of them were women (92%), and the mean age ± SD was 50 ± 12 years. BMI of the participants was 28 ± 6 kg/m^2^, and the average abdominal circumference was 92 ± 13 cm. Classic CVD risk factors were common. In this sense, 24% of the patients were current smokers, 39% had hypertension, 6% were diabetic, and 30% were obese. Likewise, 25% of the patients were taking statins and 29% were under aspirin. Besides, cIMT was 628 ± 109 µm, and carotid plaques were present in 99 (36%) SLE patients (Table 1).

**Table 1:** Characteristics of SLE patients

	SLE patients (*n* = 284)
Transforming grow factor β1, pg/ml	3164 ± 2652
Age, years	50 ± 12
Female, *n* (%)	261 (92)
Body mass index, kg/m^2^	28 ± 6
Abdominal circumference, cm	93 ± 14
Hip circumference, cm	103 ± 12
Waist-to-hip ratio	0.90 ± 0.07
Systolic pressure, mmHg	127 ± 20
Diastolic pressure, mmHg	79 ± 11
Cardiovascular co-morbidity	
Smoking, *n* (%)	69 (24)
Diabetes, *n* (%)	16 (6)
Hypertension, *n* (%)	111 (39)
Obesity, *n* (%)	85 (30)
Statins, *n* (%)	72 (25)
Aspirin, *n* (%)	80 (29)
cIMT, µm	628 ± 109
Carotid plaque, *n* (%)	99 (36)
SLE-related data	
Disease duration, years	16 (7–24)
CRP, mg/dl	2.0 (0.8–4.4)
SDI	1 (0–2)
SDI ≥ 1, *n* (%)	191 (68)
Katz Index	2 (1–4)
Katz ≥ 3, *n* (%)	126 (44)
SLEDAI	2 (0–4)
SLEDAI categories, *n* (%)	
No activity, *n* (%)	109 (40)
Mild, *n* (%)	107 (39)
Moderate, *n* (%)	41 (15)
High, *n* (%)	10 (4)
Very High, *n* (%)	4 (1)
Auto-antibody profile	
Anti-DNA positive, *n* (%)	151 (67)
ENA positive, *n* (%)	164 (69)
Anti-SSA, *n* (%)	55 (35)
Anti-SSB, *n* (%)	36 (21)
Anti-RNP, *n* (%)	64 (28)
Anti-Sm, *n* (%)	24 (10)
Anti-ribosome	13 (9)
Anti-nucleosome	32 (22)
Anti-histone	22 (15)
Antiphospholipid syndrome, *n* (%)	43 (16)
Antiphospholipid autoantibodies, *n* (%)	61 (32)
Lupus anticoagulant, *n* (%)	51 (28)
ACA IgM, *n* (%)	22 (11)
ACA IgG, *n* (%)	39 (20)
Anti beta2 glycoprotein IgM, *n* (%)	19 (10)
Anti beta2 glycoprotein IgG, *n* (%)	28 (15)
C3, mg/dl	130 ± 40
C4, mg/dl	21 ± 12
Current prednisone, *n* (%)	140 (50)
Prednisone, mg/day	5 (5–7.5)
Hydroxychloroquine, *n* (%)	194 (69)
Methotrexate, *n* (%)	31 (11)
Mycophenolate mofetil, *n* (%)	31 (11)
Azathioprine, *n* (%)	43 (15)
Rituximab, *n* (%)	8 (3)
Belimumab, *n* (%)	8 (3)

Data represent mean ± SD or median (interquartile range) when data were not normally distributed

SLEDAI categories were defined as 0, no activity; 1–5, mild; 6–10, moderate; > 10, high activity; and > 20, very high activity

*BMI* Body mass index, *C3 C4* Complement, *CRP* C-reactive protein, *DMARD* Disease-modifying antirheumatic drug, *ACA* Anticardiolipin, *ANA* Antinuclear antibodies, *ENA* Extractible nuclear antibodies, *cIMT* Carotid intima media thickness, *SLEDAI* Systemic Lupus Erythematosus Disease Activity Index, *SDI* Systemic Lupus International Collaborating Clinics/American Colleague of Rheumatology Damage Index

The disease duration was 16 (IQR 7–24) years. Most SLE patients were in the categories of no activity (40%) or mild-moderate activity (39%) as shown by the SLEDAI score. SDI and Katz indexes were median 1 (IQR 0–2) and median 2 (IQR 1–4), respectively. Seventy-eight percent of the patients had a SLICC score equal to or higher than 1. Half of the patients (50%) were taking prednisone, and the median daily dose of prednisone was 5 mg/day (IQR 5–7.5 mg). At the time of recruitment, 67% of patients were found to be positive for anti-DNA, and 69% were positive for ENA, with anti-SSA being the antibody most frequently found (35%). Sixty-nine percent of the patients were taking hydroxychloroquine when the study was performed. Other less commonly used disease-modifying antirheumatic drugs were methotrexate (11%) and azathioprine (15%). Additional information on SLE-related data is shown in Table 1. Disease characteristics relation to TGF-β1 stratified in terciles is additionally shown in Additional file 1: Table S1.

### Relationship of demographic and disease-related data with serum levels of TGF-β1

Age, abdominal circumference, waist-hip ratio, and hypertension were traditional CVD risk factors significantly associated with higher serum TGF-β1 levels (Table 2). Regarding SLE-related data, disease duration, the presence of anti-SSB, and the use of mycophenolate mofetil were associated with higher and significant circulating TGF-β1. Similarly, the Katz Severity Index, both binary (greater than or equal to 3 points) and continuous, was associated with significantly higher serum levels of TGF-β1. Besides, SLEDAI was associated with higher circulating TGF-β1. However, when this score was broken down into its different categories (no activity, mild, and moderate to very high), no difference was found between the highest compared to the no activity reference category. Moreover, the SDI score was not associated with serum levels of TGF-β1 (Table 2).

**Table 2:** Demographics and disease characteristics relation to TGF-β1 serum levels

	TGF–β1, pg/ml	
	Beta coef. 95%CI, *p*	
	Univariable	
Age, years	**28 (0.6–56)**	**0.045**
Female	− 1032 (− 2165–101)	0.074
Body mass index, kg/m^2^	46 (− 9–102)	0.10
Abdominal circumference, cm	**25 (2–48)**	**0.034**
Hip circumference, cm	7 (− 21–34)	0.64
Waist-to-hip ratio	**7726 (3479–11,972)**	** < 0.001**
Systolic pressure, mmHg	− 4 (− 20–13)	0.66
Diastolic pressure, mmHg	− 7 (− 36–22)	0.64
Cardiovascular co-morbidity		
Smoking	43 (− 688–775)	0.91
Diabetes	1300 (− 38–2639)	0.057
Hypertension	**1237 (605–1868)**	** < 0.001**
Obesity	544 (− 145–1234)	0.12
Statins	409 (− 317–1135)	0.27
Aspirin	534 (− 174–1242)	0.14
SLE-related data		
Disease duration, years	**72 (41–102)**	** < 0.001**
CRP, mg/dl	14 (− 14–41)	0.34
SDI	34 (− 146–213)	0.71
SDI ≥ 1	− 184 (− 862–495)	0.59
Katz Index	**315 (157–473)**	** < 0.001**
Katz ≥ 3	**1069 (445–1693)**	** < 0.001**
SLEDAI	**84 (8–160)**	**0.031**
SLEDAI categories		
No activity	–	–
Mild	− 46 (− 776–684)	0.90
Moderate to very high	228 (− 664–1120)	0.62
Auto-antibody profile		
Anti-DNA positive	− 416 (− 1193–361)	0.29
ENA positive	503 (− 201–1207)	0.16
Anti-SSA	428 (− 535–1390)	0.38
Anti-SSB	**2745 (584–4906)**	**0.013**
Anti-RNP	− 250 (− 1008–509)	0.52
Anti-Sm	− 465 (− 1552–622)	0.40
Anti-ribosome	− 240 (− 1649–1169)	0.74
Anti-nucleosome	828 (− 158–1814)	0.099
Anti-histone	828 (− 309–1965)	0.15
Antiphospholipid syndrome	336 (− 410–1082)	0.38
Antiphospholipid autoantibodies		
Lupus anticoagulant	648 (− 159–1456)	0.12
ACA IgM	− 181 (− 1272–910)	0.74
ACA IgG	245 (− 618–1108)	0.56
Anti beta2 glycoprotein IgM	822 (− 347–1992)	0.17
Anti beta2 glycoprotein IgG	− 28 (− 1002–946)	0.96
C3, mg/dl	− 3 (− 12–5)	0.47
C4, mg/dl	3 (− 25–31)	0.82
Current prednisone	251 (− 384–886)	0.44
Prednisone, mg/day	− 123 (− 271–25)	0.10
Hydroxychloroquine	3598 (− 1625–8821)	0.18
Methotrexate	− 651 (− 1660–358)	0.21
Mycophenolate mofetil	**1473 (445–2502)**	**0.005**
Azathioprine	50 (− 827–927)	0.91
Rituximab	− 146 (− 2022–1731)	0.88
Belimumab	816 (− 1059–2690)	0.39

In this analysis, TGF-beta serum level was considered the dependent variable

SLEDAI categories were defined as 0, no activity; 1–5, mild; 6–10, moderate; > 10, high activity; and > 20, very high activity

*BMI* Body mass index, *C3 C4* Complement, *CRP* C-reactive protein, *DMARD* Disease-modifying antirheumatic drug, *ACA* Anticardiolipin, *ANA* Antinuclear antibodies, *ENA* Extractible nuclear antibodies, *SLEDAI* Systemic Lupus Erythematosus Disease Activity Index, *SDI* Systemic Lupus International Collaborating Clinics/American Colleague of Rheumatology Damage Index. Significant beta coefficients and *p* values are depicted in bold

### Multivariable relationship of circulating TGF-β1 with lipid pattern, indices of insulin resistance, and subclinical carotid atherosclerosis

The relationship of TGF-β1 serum levels (as an independent variable) with lipid-related molecules, insulin resistance indices, and carotid subclinical atherosclerosis is shown in Table 3. In the univariable analysis, circulating TGF-β1 was positively and significantly associated with higher triglyceride levels, LDL:HDL cholesterol and apo B:apoA1 ratios, lipoprotein (a), and atherogenic index. In addition, TGF-β1 was associated with significantly lower levels of HDL cholesterol and apolipoprotein A1. These associations remained after adjustment for confounding factors, with the exception of triglycerides and lipoprotein (a). Besides, glucose homeostasis metabolism molecules and insulin resistance indices were not associated with TGF-β1 in the univariable analysis. Remarkably, TGF-β1 was associated with the presence of carotid plaque not only after adjustment for demographics (age, sex, BMI, diabetes, hypertension, and aspirin use) but also after adjustment for relationships of TGF-β1 with lipid profile molecules and insulin resistance, plus SLEDAI score (odds ratio 1.14 [95% confidence interval 1.003–1.30], *p* = 0.045) (Table 3).

**Table 3:** Multivariable analysis of the relation of TGF-β1 to lipid pattern, insulin resistance indices, and carotid subclinical atherosclerosis

		TGF-β1, ng/ml						
		Beta coef. or odds ratio (95% CI), *p*						
		Univariable		Model #1		Model #2		
Lipid profile								
Cholesterol, mg/dl	198 ± 36	− 0.2 (− 1.9 to 1.5)	0.84					
Triglycerides, mg/dl	130 ± 78	4 (0.3 to 7)	**0.032**	2 (− 1 to 6)	0.22			
LDL cholesterol, mg/dl	111 ± 29	0.3 (− 1 to 2)	0.68					
HDL cholesterol, mg/dl	61 ± 19	** − 1 (− 2 to − 0.3)**	**0.008**	** − 1 (− 2 to − 0.2)**	**0.018**			
LDL:HDL cholesterol ratio	1.96 ± 0.75	**0.04 (0.004 to 0.07)**	**0.029**	**0.04 (0.004 to 0.07)**	**0.028**			
Non-HDL cholesterol, mg/dl	137 ± 33	1 (− 0.5 to 3)	0.18	1 (0.3 to 3)	0.13			
Lipoprotein (a), mg/dl	39 (12 to 108)	**4 (0.2 to 8)**	**0.040**	4 (− 0.7 to 8)	0.10			
Apolipoprotein A1, mg/dl	173 ± 35	** − 2 (− 4 to − 0.9)**	**0.003**	** − 3 (− 4 to − 1)**	**0.002**			
Apolipoprotein B, mg/dl	95 ± 23	0.5 ( to 0.5 to 2)	0.35					
Apo B:Apo A1 ratio	0.57 ± 0.17	**0.01 (0.003 to 0.02)**	**0.004**	**0.01 (0.003 to 0.02)**	**0.005**			
Atherogenic index	3.5 ± 1.1	**0.06 (0.01 to 0.1)**	**0.016**	**0.05 (0.004 to 0.1)**	**0.035**			
Glucose homeostasis metabolism^a^								
Glucose, mg/dl	91 ± 9	− 3 (− 6 to 0.7)	0.13	− 3 (− 6 to 0.2)	0.063			
Insulin, µU/ml	6.8 (4.4 to 9.9)	− 1 (− 4 to 2)	0.43					
C-peptide, ng/ml	22 (1.5 to 3.4)	0.03 (− 0.6 to 0.6)	0.93					
HOMA2-IR	0.99 (0.65 to 1.50)	− 0.2 (− 0.6 to 0.3)	0.42					
HOMA2-S%	101 (67 to 154)	− 5 (− 20 to 10)	0.50					
HOMA2-B%-C-peptide	150 ± 88	6 (− 7 to 19)	0.37					
Carotid subclinical atherosclerosis								
cIMT, microns	628 ± 109	2 (− 3 to 7)	0.37					
Carotid plaque, *n* (%)^a^	99 (36)	**1.17 (1.06 to 1.29)**	**0.001**	**1.14 (1.02 to 1.30)**	**0.027**		**1.14 (1.003 to 1.30)**	**0.045**

In this analysis, TGF-beta serum level was considered the independent variable

Model #1 adjusted for age, sex, BMI, diabetes, hypertension, and aspirin use

Model #2 adjusted for model #1 + HDL cholesterol, lipoprotein A, apolipoprotein A1 and glucose + SLEDAI score

*LDL* Low-density lipoprotein, *HDL* High-density lipoprotein, *cIMT* Carotid intima media thickness, *TGF-β1* Transforming growth factor beta 1, *HOMA* Homeostatic model assessment, *CI* Confidence interval, *ApoA* Apolipoprotein A1, *ApoB* Apolipoprotein B

^a^Relationship of TGF-β1 to carotid plaque is shown through odds ratio (OR). Significant beta coefficients and *p* values are depicted in bold

## Discussion

Our study is the largest in the literature that addresses the relationship of TGF-β1 with subclinical atherosclerosis in patients with SLE. According to our results, patients with carotid plaque have higher circulating TGF-β1 compared to those without it. Therefore, we believe that TGF-β1 may be involved in the mechanisms that lead to CVD in patients with SLE.

Two previous studies evaluated TGF-β1 in patients with SLE. One of them recruited 70 Egyptian patients with SLE [30]. In this series, lower levels of TGF-β1 were found in patients with high disease activity (SLEDAI > 10), while the level tended to be lower in those with organ damage. In the other study that included 188 patients with SLE stratified according to the presence or absence of lupus nephritis [31], patients with nephritis showed significantly lower serum TGF-β1 values compared to patients without lupus nephritis. There were significant negative correlations between TGF-β1 levels and SLEDAI. In contrast, in our study, SLEDAI disease activity and severity indices showed a positive relationship with TGF-β1 serum levels. Unlike these previous studies, our study had a larger number of patients, focused on patients with all types of manifestations, and we measured not only disease activity but damage and severity of disease.

We believe that the inflammation or autoimmunity activity of the disease may induce the activation of TGF-β1. In this regard, the expression of TGF-β1 is context-dependent, because TGF-β can contribute to the differentiation of both suppressive and inflammatory T cells. In this sense, although TGF-β1 has anti-inflammatory and immunosuppressive effects, it is overproduced in many pathological conditions that include pulmonary fibrosis, glomerulosclerosis, renal interstitial fibrosis, cirrhosis, Crohn’s disease, cardiomyopathy, scleroderma, and chronic graft-vs-host disease [32].

Previous studies in the general population have shown that plasma levels of TGF-β1 are reduced in patients with advanced atherosclerosis and angiographically proven coronary artery disease. TGF-β1 levels were thought to be inversely related to the development and severity of coronary heart disease [14–16, 33]. In a previous work in 32 patients with SLE, low-normal TGF-β1 activation was linked with increased cIMT [34]. However, in this study, no adjustment was made for covariables due to its small sample size. In our study, TGF-β1 was positively related to the atherogenic index but negatively related to HDL and apolipoprotein A1. According to our findings, higher levels of circulating TGF-β1 would be associated with a proatherogenic profile, while lower levels of TGF-β1 would lead to a better lipid profile. In keeping with these findings, TGF-β1 was associated with the presence of carotid plaque in our cohort of SLE. We believe that the pathogenic mechanisms leading to the development of atherosclerosis in autoimmune and inflammatory diseases may differ in some way from those that occur in the general population. For example, because SLE patients have an abnormal high level of functional TGF-β1 mutations, this could lead to a secretion of defective TGF-β1 that will never activate the TGF-β1 signaling pathways after interacting with its receptor. Besides, TGF-β1 is co-secreted with latent transforming growth factor β-binding proteins and TGF-β1 propeptides [35]. Maybe, these co-secreted proteins are upregulated in SLE patients leading, therefore, to a higher detection of TGF-β1. These mechanisms may favor the upregulation of TGF-β1 in SLE, a condition predominantly related to inflammation and immune reactions.

In our study, TGF-β1 was associated with carotid plaque but not with cIMT. In this sense, cIMT and plaque may be the expression of different atherosclerosis phenotypes [36]. Moreover, ultrasound assessment of carotid plaque had higher diagnostic accuracy than the assessment of cIMT when used to predict future coronary artery events [37]. For this reason, it is possible that TGF-β1 may be related to carotid plaque and not to cIMT in patients with SLE. This may be the case in our population.

Our study has the strength that we recruited a large series of patients with SLE. We have also collected several covariables related to CVD such as a complete lipid profile. This allowed us to perform a full multivariable adjustment. We acknowledge that our cross-sectional design does not allow inferring causality. Prospective studies will be needed to test the association of TGF-β1, not only with subclinical atherosclerosis, but with CV events in this disease. We additionally acknowledge the fact that the ELISA used in our work could also recognize co-secreted propeptides that are released simultaneously with TGF-β1.

## Conclusion

In conclusion, TGF-β1 is related to the presence of carotid plaque in patients with SLE. The fact that this cytokine is associated with subclinical atherosclerosis in SLE highlights its potential role as a marker of high-risk CVD in patients with this autoimmune disease.

## Supplementary Information

**Additional file 1: Table S1.** Demographics and disease characteristics relation to TFG-beta serum levels.

## Data Availability

The data sets used and/or analyzed in the present study are available from the corresponding author upon request.

## Abbreviations

ACA: Anticardiolipin
ACVA: Acute cerebrovascular accident
ANA: Antinuclear antibodies
ApoA: Apolipoprotein A1
ApoB: Apolipoprotein B
ARA: American Rheumatism Association
BMI: Body mass index
cIMT: Carotid intima media thickness
C3 C4: Complement
CRP: C-reactive protein
CI: Confidence interval
DMARD: Disease-modifying antirheumatic drug
ENA: Extractible nuclear antibodies
HDL: High-density lipoprotein
HOMA: Homeostatic model assessment
IQR: Interquartile range
LDL: Low-density lipoprotein
SLE: Systemic lupus erythematosus
SLEDAI: Systemic Lupus Erythematosus Disease Activity Index
SDI: Systemic Lupus International Collaborating Clinics Disease Damage Index
SD: Standard deviation
TGF-β: Transforming growth factor beta

## References

[1] Li MO, Wan YY, Sanjabi S, Robertson AKL, Flavell RA. Transforming growth factor-β regulation of immune responses. Annu. Rev. Immunol. Annu Rev Immunol; 2006. p. 99–146. Available from: https://pubmed.ncbi.nlm.nih.gov/16551245/. [Cited 8 Oct 2022] 10.1146/annurev.immunol.24.021605.090737 16551245

[2] Weaver CT, Hatton RD, Mangan PR, Harrington LE. IL-17 family cytokines and the expanding diversity of effector T cell lineages. Annu Rev Immunol. Annu Rev Immunol; 2007;25:821–52. Available from: https://pubmed.ncbi.nlm.nih.gov/17201677/. [Cited 8 Oct 2022] 10.1146/annurev.immunol.25.022106.141557 17201677

[3] Prud’homme GJ. Pathobiology of transforming growth factor β in cancer, fibrosis and immunologic disease, and therapeutic considerations. Lab. Investig. Nature Publishing Group; 2007. p. 1077–91. Available from: https://www.nature.com/articles/3700669. [Cited 8 Oct 2022] 10.1038/labinvest.3700669 17724448

[4] Schoenfeld SR, Kasturi S, Costenbader KH. The epidemiology of atherosclerotic cardiovascular disease among patients with SLE: a systematic review. Semin Arthritis Rheum. Semin Arthritis Rheum; 2013;43:77–95. Available from: https://pubmed.ncbi.nlm.nih.gov/23422269/. [Cited 23 Oct 2022] 10.1016/j.semarthrit.2012.12.002 23422269

[5] Quevedo-Abeledo JC, Rúa-Figueroa Í, Sánchez-Pérez H, Tejera-Segura B, De Vera-González A, González-Delgado A (2019). Disease damage influences cardiovascular risk reclassification based on carotid ultrasound in patients with systemic lupus erythematosus. J Rheumatol.

[6] Martín-González C, Ferrer-Moure C, Quevedo-Abeledo JC, González-Gay MÁ, Ferraz-Amaro I. Apolipoprotein C3 and beta-cell dysfunction are linked in patients with systemic lupus erythematosus. Clin Exp Rheumatol. 2021; Available from: http://www.ncbi.nlm.nih.gov/pubmed/34936546. [Cited 23 Oct 2022] 10.55563/clinexprheumatol/cezjnr 34936546

[7] Martín-González C, Ferrer-Moure C, Carlos Quevedo-Abeledo J, de Vera-González A, González-Delgado A, Sánchez-Martín J, et al. Apolipoprotein C-III in patients with systemic lupus erythematosus. Arthritis Res Ther. BioMed Central Ltd; 2022;24. 10.1186/s13075-022-02793-y PMC9088095 35538496

[8] Sánchez-Pérez H, Quevedo-Abeledo JC, De Armas-Rillo L, Rua - Figueroa Í, Tejera-Segura B, Armas-González E (2020). Impaired HDL cholesterol efflux capacity in systemic lupus erythematosus patients is related to subclinical carotid atherosclerosis. Rheumatol (United Kingdom). Oxford University Press.

[9] Sánchez-Pérez H, Quevedo-Abeledo JC, Tejera-Segura B, de Armas-Rillo L, Rúa-Figueroa I, González-Gay MA, et al. Proprotein convertase subtilisin/kexin type 9 is related to disease activity and damage in patients with systemic erythematosus lupus. Ther Adv Musculoskelet Dis. SAGE Publications Ltd; 2020;12:1759720X20975904. Available from: http://www.ncbi.nlm.nih.gov/pubmed/33294038. [Cited 23 Oct 2022] 10.1177/1759720X20975904 PMC7705780 33294038

[10] Quevedo-Abeledo JC, Martín-González C, Ferrer-Moure C, de Armas-Rillo L, Hernandez-Hernandez MV, González-Gay M, et al. Key molecules of triglycerides pathway metabolism are disturbed in patients with systemic lupus erythematosus. Front Immunol. 2022;13:827355. 10.3389/fimmu.2022.827355 PMC9124762 35615358

[11] Quevedo-Abeledo JC, Hernandez-Díaz M, Sánchez-Perez H, Medina-Vega L, González-Rivero AF, Almeida-Santiago C, et al. Amylin serum levels are upregulated in patients with systemic lupus erythematosus. Clin Exp Rheumatol. 2022;40:1378–84. 10.55563/clinexprheumatol/h5cidq 34596033

[12] García-Dorta A, Quevedo-Abeledo JC, Rua-Figueroa Í, de Vera-González AM, González-Delgado A, Medina-Vega L, et al. Beta-cell function is disrupted in patients with systemic lupus erythematosus. Rheumatology (Oxford) 2021;60:3826–33. 10.1093/rheumatology/keaa874 33369681

[13] Martín-González C, Ferrer-Moure C, Quevedo-Abeledo JC, González-Gay MÁ, Ferraz-Amaro I. Apolipoprotein C3 and beta-cell dysfunction are linked in patients with systemic lupus erythematosus. Clin Exp Rheumatol. 2021;40:2032–37. 10.55563/clinexprheumatol/cezjnr 34936546

[14] Batuman O, Go D, Clark LT, Smith ELP, Clements P, Feit A, et al. Relationship between cytokine levels and coronary artery disease in women. Heart Dis. Heart Dis. 2001;3:80–4. Available from: https://pubmed.ncbi.nlm.nih.gov/11975775/. 10.1097/00132580-200103000-00004 11975775

[15] Li CG, Bethell H, Wilson PB, Bhatnagar D, Walker MG, Kumar S. The significance of CD105, TGFbeta and CD105/TGFbeta complexes in coronary artery disease. Atherosclerosis. Atherosclerosis; 2000;152:249–56. Available from: https://pubmed.ncbi.nlm.nih.gov/10996361/. [Cited 23 Oct 2022] 10.1016/s0021-9150(99)00476-1 10996361

[16] Grainger DJ, Kemp PR, Metcalfe JC, Liu AC, Lawn RM, Williams NR, et al. The serum concentration of active transforming growth factor-beta is severely depressed in advanced atherosclerosis. Nat Med. Nat Med; 1995;1:74–9. Available from: https://pubmed.ncbi.nlm.nih.gov/7584958/. [Cited 23 Oct 2022] 10.1038/nm0195-74 7584958

[17] Ten Dijke P, Arthur HM. Extracellular control of TGFβ signalling in vascular development and disease. Nat. Rev. Mol. Cell Biol. Nat Rev Mol Cell Biol; 2007. p. 857–69. Available from: https://pubmed.ncbi.nlm.nih.gov/17895899/. [Cited 23 Oct 2022] 10.1038/nrm2262 17895899

[18] Cruz-González D de J, Gómez-Martin D, Layseca-Espinosa E, Baranda L, Abud-Mendoza C, Alcocer-Varela J, et al. Analysis of the regulatory function of natural killer cells from patients with systemic lupus erythematosus. Clin Exp Immunol. Clin Exp Immunol; 2018 [cited 2022 Oct 9];191:288–300. Available from: https://pubmed.ncbi.nlm.nih.gov/29058308/. [Cited 2022 Oct 9] 10.1111/cei.13073 PMC5801498 29058308

[19] Ohtsuka K, Gray JD, Stimmler MM, Toro B, Horwitz DA. Decreased production of TGF-beta by lymphocytes from patients with systemic lupus erythematosus. J Immunol [Internet]. 1998 [cited 2022 Oct 12];160:2539–45. Available from: https://pubmed.ncbi.nlm.nih.gov/9498800/9498800

[20] Ohtsuka K, Gray JD, Quismorio FP, Lee W, Horwitz DA. Cytokine-mediated down-regulation of B cell activity in SLE: effects of interleukin-2 and transforming growth factor-beta. Lupus. Lupus; 1999 [cited 2022 Oct 12];8:95–102. Available from: https://pubmed.ncbi.nlm.nih.gov/10192502/. [Cited 12 Oct 2022] 10.1191/096120399678847498 10192502

[21] Hochberg MC. Updating the American College of Rheumatology revised criteria for the classification of systemic lupus erythematosus. Arthritis Rheum. 1997;40:1725. Available from: http://www.ncbi.nlm.nih.gov/pubmed/9324032. [Cited 30 May 2019] 10.1002/art.1780400928 9324032

[22] Wallace TM, Levy JC, Matthews DR. Use and abuse of HOMA modeling. Diabetes Care. 2004;27:1487–95. Available from: http://www.ncbi.nlm.nih.gov/pubmed/15161807. [Cited 9 Mar 2019] 10.2337/diacare.27.6.1487 15161807

[23] Gladman DD, Ibañez D, Urowltz MB. Systemic Lupus Erythematosus Disease Activity Index 2000. J Rheumatol. 2002;29:288–91. Available from: http://www.ncbi.nlm.nih.gov/pubmed/11838846. [Cited 10 Nov 2018] 11838846

[24] Gladman D, Ginzler E, Goldsmith C, Fortin P, Liang M, Urowitz M, et al. The development and initial validation of the Systemic Lupus International Collaborating Clinics/American College of Rheumatology damage index for systemic lupus erythematosus. Arthritis Rheum. 1996;39:363–9. Available from: http://www.ncbi.nlm.nih.gov/pubmed/8607884. [Cited 10 Nov 2018] 10.1002/art.1780390303 8607884

[25] Mosca M, Bombardieri S. Assessing remission in systemic lupus erythematosus. Clin Exp Rheumatol.;24:S-99–104. Available from: http://www.ncbi.nlm.nih.gov/pubmed/17083771. [Cited 30 May 2019] 17083771

[26] Katz JD, Senegal J-L, Rivest C, Goulet J-R, Rothfield N. A simple severity of disease index for systemic lupus erythematosus. Lupus. 1993;2:119–23. Available from: http://www.ncbi.nlm.nih.gov/pubmed/8330033. [Cited 30 May 2019] 10.1177/096120339300200210 8330033

[27] Tejera-Segura B, Macía-Díaz M, Machado JD, de Vera-González A, García-Dopico JA, Olmos JM, et al. HDL cholesterol efflux capacity in rheumatoid arthritis patients: contributing factors and relationship with subclinical atherosclerosis. Arthritis Res Ther. 2017;19:113. Available from: http://arthritis-research.biomedcentral.com/articles/10.1186/s13075-017-1311-3. [Cited 10 Jun 2019] 10.1186/s13075-017-1311-3 PMC5452399 28569219

[28] Corrales A, González-Juanatey C, Peiró ME, Blanco R, Llorca J, González-Gay MA. Carotid ultrasound is useful for the cardiovascular risk stratification of patients with rheumatoid arthritis: results of a population-based study. Ann Rheum Dis. 2014;73:722–7. Available from: http://ard.bmj.com/lookup/doi/10.1136/annrheumdis-2012-203101. [Cited 30 May 2019] 10.1136/annrheumdis-2012-203101 23505241

[29] Touboul P-J, Hennerici MG, Meairs S, Adams H, Amarenco P, Bornstein N, et al. Mannheim carotid intima-media thickness consensus (2004–2006). An update on behalf of the Advisory Board of the 3rd and 4th Watching the Risk Symposium, 13th and 15th European Stroke Conferences, Mannheim, Germany, 2004, and Brussels, Belgium, 2006. Cerebrovasc Dis. 2007;23:75–80. Available from: https://www.karger.com/Article/FullText/97034. [Cited 30 May 2019] 10.1159/000097034 17108679

[30] Metawie SA, ElRefai RM, ElAdle SS, Shahin RMH (2015). Transforming growth factor-β1 in systemic lupus erythematosus patients and its relation to organ damage and disease activity. Egypt Rheumatol Elsevier.

[31] Rashad NM, El-Shabrawy RM, Said D, El-Shabrawy SM, Emad G. Serum levels of transforming growth factor beta-1 (TGF-β1) as an early no invasive marker for diagnosis of lupus nephritis in systemic lupus erythematosus patients. Egypt J Immuno. 2019;26:31–42. Available from: https://pubmed.ncbi.nlm.nih.gov/31332994/. [Cited 11 Oct 2022] 31332994

[32] Prud’homme GJ. Pathobiology of transforming growth factor β in cancer, fibrosis and immunologic disease, and therapeutic considerations. Lab. Investig. Nature Publishing Group; 2007. p. 1077–91. Available from: https://www.nature.com/articles/370066. [Cited 12 Oct 2022] 10.1038/labinvest.3700669 17724448

[33] Chen C, Lei W, Chen W, Zhong J, Gao X, Li B, et al. Serum TGF-β1 and SMAD3 levels are closely associated with coronary artery disease. BMC Cardiovasc Disord. BioMed Central Ltd.; 2014;14:1–7. Available from: https://bmccardiovascdisord.biomedcentral.com/articles/10.1186/1471-2261-14-18. [Cited 23 Oct 2022] 10.1186/1471-2261-14-18 PMC3936998 24533640

[34] Jackson M, Ahmad Y, Bruce IN, Coupes B, Brenchley PE. Activation of transforming growth factor-β1 and early atherosclerosis in systemic lupus erythematosus. Arthritis Res Ther. 2006;8. Available from: http://arthritis-research.com/content/8/3/R81Thisarticleisonlineat. [Cited 24 Oct 2022] 10.1186/ar1951 PMC1526639 16646981

[35] Robertson IB, Horiguchi M, Zilberberg L, Dabovic B, Hadjiolova K, Rifkin DB. Latent TGF-β-binding proteins. Matrix Biol. NIH Public Access; 2015:44–53. 10.1016/j.matbio.2015.05.005 PMC4844006 25960419

[36] Spence D. Measurement of intima-media thickness vs. carotid plaque: uses in patient care, genetic research and evaluation of new therapies. Int. J. Stroke. Int J Stroke; 2006. p. 216–21. Available from: https://pubmed.ncbi.nlm.nih.gov/18706019/. [Cited 23 Oct 2022] 10.1111/j.1747-4949.2006.00068.x 18706019

[37] Simon A, Megnien JL, Chironi G. The value of carotid intima-media thickness for predicting cardiovascular risk. Arterioscler. Thromb. Vasc. Biol. Lippincott Williams & Wilkins; 2010. p. 182–5. Available from: https://www.ahajournals.org/doi/abs/10.1161/ATVBAHA.109.196980. [Cited 23 Oct 2022] 10.1161/ATVBAHA.109.196980 19948842

