# Supplementary material for: Transforming growth factor beta 1 is associated with subclinical carotid atherosclerosis in patients with systemic lupus erythematosus
Source: Arthritis Res Ther. 2023 Apr 17;25:64. doi: 10.1186/s13075-023-03046-2 (PMC10108540; doi:10.1186/s13075-023-03046-2)
Supplement: Supplementary file 1 — Additional file 1: Table S1. Demographics and disease characteristics relation to TFG-beta serum levels. [file 13075_2023_3046_MOESM1_ESM.docx]

| **Supplementary Table 1. Demographics and disease characteristics relation to TFG-beta serum levels** | | | | | | | | |  |
| --- | --- | --- | --- | --- | --- | --- | --- | --- | --- |
|  |  | TGF-β1, ng/ml | | 3rd tertile TGF-β1, ng/ml | |  |  |  |  |
|  |  | beta coef. (95%CI), p | | OR (95%CI), p | |  |  |  |  |
| Age, years | | **28 (0.6-56)** | **0.045** | **1.03 (1.003-1.06)** | **0.032** |  |  |  |  |
| Female | | -1032 (-2165-101) | 0.074 | 0.41 (0.14-1.24) | 0.12 |  |  |  |  |
| Body mass index, kg/m2 | | 46 (-9-102) | 0.10 | 1.05 (0.99-1.10) | 0.10 |  |  |  |  |
| Abdominal circumference, cm | | **25 (2-48)** | **0.034** | 1.02 (0.99-1.04) | 0.056 |  |  |  |  |
| Hip circumference, cm | | 7 (-21-34) | 0.64 | 1.00 (0.98-1.03) | 0.79 |  |  |  |  |
| Waist-to-hip ratio | | **7726 (3479-11972)** | **<0.001** | **1323 (18-99683)** | **0.001** |  |  |  |  |
| Sytolic pressure, mmHg | | -4 (-20-13) | 0.66 | 1.00 (0.98-1.01) | 0.77 |  |  |  |  |
| Diastolic pressure, mmHg | | -7 (-36-22) | 0.64 | 0.97 (0.95-1.00) | 0.052 |  |  |  |  |
| Cardiovascular co-morbidity | |  |  |  |  |  |  |  |  |
| Smoking | | 43 (-688-775) | 0.91 | 1.30 (0.66-2.54) | 0.44 |  |  |  |  |
| Diabetes | | 1300 (-38-2639) | 0.057 | 1.35 (0.48-3.79) | 0.57 |  |  |  |  |
| Hypertension | | **1237 (605-1868)** | **<0.001** | 2.29 (1.25-4.20) | 0.008 |  |  |  |  |
| Obesity | | 544 (-145-1234) | 0.12 | **2.22 (1.14-4.29)** | **0.018** |  |  |  |  |
| Statins | | 409 (-317-1135) | 0.27 | 1.54 (0.79-3.00) | 0.21 |  |  |  |  |
| Aspirin | | 534 (-174-1242) | 0.14 | 1.62 (0.85-3.09) | 0.14 |  |  |  |  |
| SLE related data | |  |  |  |  |  |  |  |  |
| Disease duration, years | | **72 (41-102)** | **<0.001** | **1.05 (1.02-1.08)** | **0.001** |  |  |  |  |
| CRP, mg/dl | | 14 (-14-41) | 0.34 | 1.02 (0.98-1.05) | 0.30 |  |  |  |  |
| SLICC | | 34 (-146-213) | 0.71 | 1.01 (0.86-1.19) | 0.87 |  |  |  |  |
| SLICC >=1 | | -184 (-862-495) | 0.59 | 0.72 (0.39-1.34) | 0.30 |  |  |  |  |
| Katz Index | | **315 (157-473)** | **<0.001** | **1.27 (1.08-1.49)** | **0.003** |  |  |  |  |
| Katz >=3 | | **1069 (445-1693)** | **<0.001** | **2.55 (1.40-4.64)** | **0.002** |  |  |  |  |
| SLEDAI | | **84 (8-160)** | **0.031** | 1.08 (0.99-1.17) | 0.062 |  |  |  |  |
| SLEDAI categories | |  |  |  |  |  |  |  |  |
|  | No activity | - | - | - | - |  |  |  |  |
|  | Mild | -46 (-776-684) | 0.90 | 1.18 (0.61-2.28) | 0.62 |  |  |  |  |
|  | Moderate to very high | 228 (-664-1120) | 0.62 | 1.64 (0.72-3.73) | 0.24 |  |  |  |  |
| Auto-antibody profile | |  |  |  |  |  |  |  |  |
|  | Anti-DNA positive | -416 (-1193-361) | 0.29 | **0.40 (0.19-0.86)** | **0.019** |  |  |  |  |
|  | ENA positive | 503 (-201-1207) | 0.16 | 1.49 (0.72-3.07) | 0.29 |  |  |  |  |
|  | Anti-SSA | 428 (-535-1390) | 0.38 | 1.33 (0.59-2.96) | 0.49 |  |  |  |  |
|  | Anti-SSB | **2745 (584-4906)** | **0.013** | 5.67 (0.66-48.83) | 0.11 |  |  |  |  |
|  | Anti-RNP | -250 (-1008-509) | 0.52 | 0.67 (0.32-1.38) | 0.28 |  |  |  |  |
|  | Anti-Sm | -465 (-1552-622) | 0.40 | 0.48 (0.14-1.65) | 0.25 |  |  |  |  |
|  | Anti-ribosome | -240 (-1649-1169) | 0.74 | 0.39 (0.10-1.61) | 0.20 |  |  |  |  |
|  | Anti-nucleosome | 828 (-158-1814) | 0.099 | 2.46 (0.94-6.41) | 0.066 |  |  |  |  |
|  | Anti-histone | 828 (-309-1965) | 0.15 | 2.83 (0.92-8.73) | 0.070 |  |  |  |  |
| Antiphospholipid syndrome | | 336 (-410-1082) | 0.38 | 1.88 (0.83-4.22) | 0.13 |  |  |  |  |
| Antiphospholipid autoantibodies | |  |  |  |  |  |  |  |  |
|  | Lupus anticoagulant | 648 (-159-1456) | 0.12 | 1.92 (0.80-4.63) | 0.15 |  |  |  |  |
|  | ACA IgM | -181 (-1272-910) | 0.74 | 1.00 (0.31-3.27) | 0.99 |  |  |  |  |
|  | ACA IgG | 245 (-618-1108) | 0.56 | 1.34 (0.55-3.25) | 0.52 |  |  |  |  |
|  | Anti beta2 glycoprotein IgM | 822 (-347-1992) | 0.17 | 2.95 (0.90-9.64) | 0.074 |  |  |  |  |
|  | Anti beta2 glycoprotein IgG | -28 (-1002-946) | 0.96 | 1.15 (0.40-3.27) | 0.80 |  |  |  |  |
| C3, mg/dl | | -3 (-12-5) | 0.47 | 0.99 (0.99-1.00) | 0.14 |  |  |  |  |
| C4, mg/dl | | 3 (-25-31) | 0.82 | 1.00 (0.98-1.03) | 0.93 |  |  |  |  |
| Current prednisone | | 251 (-384-886) | 0.44 | 1.56 (0.87-2.79) | 0.14 |  |  |  |  |
| Prednisone, mg/day | | -123 (-271-25) | 0.10 | 0.93 (0.82-1.06) | 0.28 |  |  |  |  |
| Hydroxychloroquine | | 3598 (-1625-8821) | 0.18 | **0.40 (0.21-0.77)** | **0.006** |  |  |  |  |
| Methotrexate | | -651 (-1660-358) | 0.21 | 0.83 (0.34-2.04) | 0.69 |  |  |  |  |
| Mycophenolate mofetil | | **1473 (445-2502)** | **0.005** | 2.93 (1.00-8.60) | 0.050 |  |  |  |  |
| Azathioprine | | 50 (-827-927) | 0.91 | 1.11 (0.50-2.46) | 0.79 |  |  |  |  |
| Rituximab | | -146 (-2022-1731) | 0.88 | 0.76 (0.16-3.49) | 0.72 |  |  |  |  |
| Belimumab | | 816 (-1059-2690) | 0.39 | 2.65 (0.50-14.00) | 0.25 |  |  |  |  |
| In this analysis TGF-beta serum levels are considered the dependent variable. | | | | | | | | |  |
| BMI: body mass index; C3 C4: complement; CRP: C reactive protein. | | | | | | | |  |  |
| DMARD: disease-modifying antirheumatic drug; ACA: anticardiolipin. | | | | | | | | |  |
| ANA: antinuclear antibodies; ENA: extractible nuclear antibodies. | | | | | | | |  |  |
| SLEDAI: Systemic Lupus Erythematosus Disease Activity Index. | | | | | | | |  |  |
| SLEDAI categories were defined as: 0, no activity; 1-5 mild; 6-10 moderate; >10 high activity,  >20 very high activity. | | | | | | | | | |
| SLICC: Systemic Lupus International Collaborating Clinics/American Colleague of Rheumatology  Damage Index. | | | | | | | | | |
| For the analysis of categorical SLEDAI to 3rd tertil of TGF-β1, only the results of 3rd category of  SLEDAI against remission is shown. Significant beta coefficients and p values are depicted in bold. | | | | | | |  |  |  |
